# Regulation of potassium homeostasis in *Mycoplasma bovis* by the diadenylate cyclase CdaM

**DOI:** 10.3389/fmicb.2026.1757129

**Published:** 2026-03-13

**Authors:** Jiongxi Chen, Doukun Lu, Yingjie Fu, Tahira Iftakhar, Zhiyu Hao, Menghan Zhang, Xi Chen, Yingyu Chen, Changmin Hu, Jianguo Chen, Lei Zhang, Gang Zhao, Aizhen Guo

**Affiliations:** 1 National Key Laboratory of Agricultural Microbiology, Huazhong Agricultural University, Wuhan, China; 2 Hubei Hongshan Laboratory, Huazhong Agricultural University, Wuhan, China; 3 College of Veterinary Medicine, Huazhong Agricultural University, Wuhan, China; 4 International Research Center for Animal Disease, Ministry of Science and Technology, Huazhong Agricultural University, Wuhan, China; 5 Hubei International Scientific and Technological Cooperation Base of Veterinary Epidemiology, Huazhong Agricultural University, Wuhan, China; 6 Key Laboratory of Ministry of Education for Conservation and Utilization of Special Biological Resources in the Western China, School of Life Sciences, Ningxia University, Yinchuan, China

**Keywords:** c-di-AMP, diadenylate cyclase, K^+^ homeostasis, K^+^ transporter, MbovP496, CdaM, *Mycoplasma*, *Mycoplasma bovis*

## Abstract

Cyclic di-AMP (c-di-AMP) is a critical second messenger in many Gram-positive bacteria and archaea that regulates intracellular potassium (K^+^) concentrations, ensuring osmotic balance. However, the precise mechanisms of K^+^ regulation by c-di-AMP in *Mycoplasma* species remain largely unexplored. In this study, we used the ruminant pathogen *Mycoplasma bovis* (*M. bovis*) as a model to investigate this mechanism. We identified CdaM (MbovP496), a member of the DisA_N family, a member of the DisA_N family, as a functional diadenylate cyclase capable of synthesizing c-di-AMP, and demonstrated that its enzymatic activity depends on the conserved DGA and RHR motifs. Deletion of *cdaM* (cdaM*) abolished c-di-AMP production and resulted in pronounced growth defects when *M. bovis* was co-cultured with host cells or grown in PPLO medium supplemented with exogenous K^+^. These phenotypes were accompanied by increased expression of the K^+^ uptake transporter TrkA (MbovP421). We further demonstrated that TrkA acts as a direct receptor for c-di-AMP and identified the residues R174, V180, and P192 as critical for this interaction. Loss of CdaM resulted in significantly elevated intracellular K^+^ levels, underscoring the essential role of c-di-AMP in maintaining K^+^ homeostasis. Transcriptomic analysis further revealed that genes differentially expressed between the wild type strain and cdaM* mutant were enriched in pathways related to transmembrane transport and pyruvate metabolism, indicating broader metabolic reprogramming associated with disrupted c-di-AMP signaling. In conclusion, this study identifies CdaM as a key determinant of K^+^ adaptation in *M. bovis* and elucidates the molecular interaction between its product and the K^+^ uptake transporter TrkA. Together, these findings provide important insights into c-di-AMP-mediated regulation of intracellular K^+^ concentration in mycoplasmas and highlight DisA_N family proteins as potential targets for antimicrobial intervention.

## Introduction

As the smallest prokaryote capable of independent replication (Citti and Blanchard, 2013), *Mycoplasma* poses a significant concern due to its pathogenicity in humans and various animal species, causing diseases such as pneumonia, vaginitis, mastitis, and osteoarthritis (Waites and Talkington, 2004; Tsevat et al., 2017; Fox, 2012; Gourlay, 1981). These organisms are members of the *Mollicutes* class (Trachtenberg, 1998), which is a broad category of wall-less bacteria, and has undergone a significant reduction in genome size throughout their evolution from Gram-positive progenitors (Razin et al., 1998). Their small genome has provided researchers with a useful platform to investigate important biological issues. Moreover, *Mycoplasmas* are adept at enduring environmental stresses like heat and drying and exhibit increased incidences of antibiotic resistance and immune evasion (Chernova et al., 2016; Askar et al., 2021). Therefore, a deeper understanding of their physiology is necessary to effectively combat associated diseases.

For adaptation to dynamic settings, bacteria require effective mechanisms that convert various stimuli into information signals, enabling them to perceive these alterations and react accordingly. The production of second messengers is a crucial mechanism for adaptation (Gomelsky, 2011; Whiteley et al., 2015). Cyclic di–adenosine monophosphate (c-di-AMP) is an important bacterial second messenger found in Gram-positive and mycobacteria (Zheng et al., 2014), it is generated by the catalysis of two ATP molecules in the presence of diadenylate cyclase (DAC) (Witte et al., 2008; Bai et al., 2012) and is degraded into either phosphoadenylyl adenine (pApA) or AMP in the presence of phosphodiesterase (PDE) (Bai et al., 2013; Rao et al., 2010). c-di-AMP-specific DACs are present in a wide range of bacteria and play an important role in maintaining multiple physiological functions, including biofilm formation (Kaplan Zeevi et al., 2013; Witte et al., 2013), cell size regulation (Corrigan et al., 2011), toxicity (Ye et al., 2014; Du et al., 2014), ion transport homeostasis (Bai et al., 2014), and antibiotics resistance, among others (Nolan et al., 2023; Pham et al., 2021). In 2010, c-di-AMP was found to be a secreted molecule able to trigger the cytosolic host response and overexpression of the DAC can result in elevated levels of the host response during infection (Woodward et al., 2010). Later, DACs have been more intensively investigated as a promising antimicrobial drug and anti-virulence target in a variety of bacteria (Kalia et al., 2013; Opoku-Temeng et al., 2016).

As environmental inputs can regulate the levels of c-di-AMP, it has been suggested to be a central regulator of the machinery responsible for maintaining K^+^ homeostasis (Gundlach et al., 2018; Gundlach et al., 2017a,b; Zarrella et al., 2018), inhibiting the transit of K^+^ (Bai et al., 2014; Corrigan et al., 2013; Albright et al., 2006; Zeden et al., 2018), and finally affect cell integrity (Commichau et al., 2018; Foster et al., 2024). For instance, c-di-AMP attaches to and inhibits the related Kup K^+^ transporters in *Lactococcus lactis* (Quintana et al., 2019). Moreover, c-di-AMP binds to the KdpD sensor kinase, regulating the activity of the Kdp K^+^ transport systems in *Staphylococcus aureus* and *Listeria monocytogene* (Moscoso et al., 2016). Therefore, controlling the synthesis of c-di-AMP is particularly important for regulating the bacterial K^+^ homeostasis. In *Mycoplasma pneumoniae*, a c-di-AMP cyclase CdaM (MPN244) was identified and its product c-di-AMP can bind to the cytoplasmic regulatory subunit KtrC of the K^+^ transporter (Blötz et al., 2017). However, this study did not elaborate on the combined effects of these interactions in *Mycoplasma pneumoniae*. Despite growing interest in this area, the specific mechanisms underlying K^+^ regulation in *Mycoplasma* remain insufficiently characterized.

In a prior study, we employed a transposon mutant library and a co-culture model to uncover two *M. bovis* mutants exhibiting growth defects (Zhu et al., 2020a). These mutants encode the phosphodiesterases MbovP328 and MbovP276, which are involved in the degradation of c-di-AMP and play crucial role in cyclic dinucleotide regulation (Zhu et al., 2020a; Zhu et al., 2023). To further understand the dynamics of c-di-AMP, we used *M. bovis* as a model organism and conducted a genome-wide sequence comparison with known c-di-AMP cyclases. This analysis led to the identification of a prototypical DisA_N family diadenylate cyclase, CdaM (MbovP496). Phenotypic characterization of the cdaM mutant revealed its critical role in maintaining K^+^ homeostasis and supporting osmotic tolerance in *M. bovis*.

## Methods and materials

### Ethics statement

The protocol for the animal experiment (HZAUMO-2024-0110), aimed at generating mouse antiserum, underwent a thorough review and subsequently received approval from the Animal Ethics Committee of Huazhong Agricultural University, located in Wuhan, China. All experimental procedures were carried out in strict adherence to the “Guide for the Care and Use of Laboratory Animals” as stipulated in Hubei Province, China, and were documented in full compliance with the ARRIVE guidelines.

### Growth of bacterial strains and cells

*M. bovis* HB0801 (CCTCC No. M2010040), originally isolated in 2008 from Hubei, China, was cultured in pleuropneumonia-like organism (PPLO) medium (BD, MD, United States) at 37 °C, as described previously (Qi et al., 2012). The *M. bovis* mutant cdaM* (T5.415) and its corresponding complementary strain cdaM*::p*cdaM* (CT5.415) were both cultured in PPLO medium. Specifically, the medium for cdaM* was supplemented with 100 μg/mL of gentamicin, while the medium for cdaM*::p*cdaM* was augmented with 10 μg/mL puromycin. *Mycoplasma* colony-forming units (CFUs) were determined as previously described (Baranowski et al., 2010). *Escherichia coli* strains DH5α and BL21 (TransGen Biotech, Beijing, China) were grown in Luria-Bertani broth (LB) with 30 μg/mL kanamycin. EBL cells were cultured in minimum essential medium (MEM) containing 10% heat-inactivated fetal calf serum (Gibco, NY, United States).

### Bioinformatics analysis

The potential c-di-AMP cyclase of *M. bovis* was predicted by using BLASTP,^1^ and the conserved domains were analyzed with the NCBI Conserved Domain Database.^2^ The homology motifs of CdaM (MbovP496) and other seven bacterial species (*Listeria monocytogenes*, *Paenibacillus mucilaginosus*, *Thermobacillus xylanilyticus*, *Streptococcus pneumoniae*, *Mycobacterium tuberculosis*, *Staphylococcus aureus,* and *Mycoplasma pneumoniae*) were generated with ClustalW for multiple sequence alignment^3^ and ESPript.^4^

Pfam was used to predict the conserved functional domains of K^+^ transporters MbovP421 and MbovP415. The highly homologous K^+^ transporter MbovP421 from the TrkA family in *M. bovis* was retrieved from the RCSB PDB: Homepage of the Protein Data Bank,^5^ and the small molecule c-di-AMP was obtained from PubChem.^6^ The small molecule structure was optimized using the MMFF94 force field in OpenBabel 3.1.1 software to obtain the lowest energy conformation. AutoDock Tools 1.5.6 was used for protein and small molecule hydrogenation, as well as torsion detection (Forli et al., 2016). The molecular docking parameters were configured utilizing the Grid module (within the relevant software/platform). The docking procedure employed a semi-flexible approach, characterized by an exhaustiveness setting of 25. The docking results were analyzed and visualized by AutoDock Vina 1.2.0 and PyMOL 2.3.0 software, respectively (Morris et al., 2009; Zhang et al., 2022). Predicted interaction residues between c-di-AMP and TrkA (MbovP421) were identified based on docking-derived binding pocket analysis.

### DNA manipulation

The cdaM* (T5.415) strain with mutation at nucleotide (nt) position 280 within the *cdaM* (*Mbov_0496*) coding sequence (CDS), was identified from transposon-mutagenized library by PCR (Zhu et al., 2020b). Plasmid pOH/P was used to construct the *M. bovis cdaM* complementary strain using a previously described method (Baranowski et al., 2018). The *cdaM* coding sequence (CDS) was synthesized together with the upstream promoter sequence of *M. agalactiae* P40 (Baranowski et al., 2010) by Beijing Tianyi Huiyuan Bioscience & Technology Inc. (Wuhan, China), and subsequently cloned into the pOH/P plasmid, which was named pOH/P-*cdaM*. The cdaM* strain was then transfected with pOH/P-*cdaM* to generate the complementary strain, cdaM*::p*cdaM* (CT5.415) (Zhao et al., 2021).

For *M. bovis* protein expression in *Escherichia coli*, the *cdaM* sequence was modified and synthesized. For modification, the UGA tryptophan codon in *M. bovis* was changed to UGG to avoid premature stop codons in *E. coli*. The DNA sequences encoding CdaM mutants (CdaM^D110A/G111A^, CdaM^R141A/H142A/R143A^, and CdaM^D110A/G111A/R141A/H142A/R143A mutants^) and TrkA mutants (TrkA^R174A^, TrkA^V180A^, TrkA^V181A^, TrkA^L191A^, TrkA^P192A^, TrkA^S193A^, and TrkA^I207A^) were cloned into pET-30a plasmid. The above residues were changed to alanine by overlap extension PCR. The oligonucleotide primers used for DNA construction in this study are listed in Supplementary Table S1.

### Expression and purification of *Mycoplasma bovis* recombinant proteins

The recombinant pET-30a plasmids were transformed into *E. coli* BL21 cells for protein expression, as previously described (Zhu et al., 2020b). Briefly, protein expression was induced with 0.8 mM isopropyl-β-D-thiogalactopyranoside (IPTG) at 16 °C for 24 h. Cultures were then collected by centrifugation, resuspended in lysis buffer (20 mM Na_3_PO_4_, 0.5 M NaCl, 5 mM imidazole, pH 7.4), and sonicated at 4 °C under 1,000 bar for 3 times. Soluble proteins were purified using nickel affinity chromatography (GE Healthcare, Piscataway, NJ, United States) after centrifugation at 10,000 × g for 30 min. Purified proteins were analyzed by SDS-PAGE, and protein concentrations were determined by BCA protein assay (Beyotime Biotechnology, Shanghai, China).

### Enzymatic activity assay

The c-di-AMP synthesis activities of the recombinant protein rCdaM and its mutants (rCdaM^D110A/G111A^, rCdaM^R141A/H142A/R143A^, and rCdaM^D110A/G111A/R141A/H142A/R143A^) were assessed using an enzymatic activity assay. To assess the cyclase activity of rCdaM and its catalytic sites, the assays were performed at a temperature of 37 °C in a buffer solution containing 100 mM HEPES (pH 7.0), 2 mM ATP or ADP (Yuan Ye Science Institute, Shanghai, China), 10 mM MgCl_2_, and 1 mM recombinant protein (10 μM rCdaM was used to determine the optimal enzymatic reaction conditions). The reaction was terminated by boiling for 10 min, and insoluble materials were removed by centrifugation at 20,000 × g followed by filtration through a 0.22 μm filter. The c-di-AMP in the soluble fraction was analyzed by high-performance liquid chromatography (HPLC) (Shimadzu Corporation, Kyoto, Japan) using an RP-C18 column (4.6 × 250 mm, 5 μm; Thermo Fisher Scientific, MA, United States) as previously described (Zheng et al., 2013). Optimal reaction temperature, metal ion preference, Mn^2+^ concentration, and pH dependence were determined using the above method.

### Growth curves of *Mycoplasma bovis*

Serial gradient dilutions of *M. bovis* WT, cdaM*, and cdaM*::p*cdaM* strains were prepared and adjusted to a final concentration of 10^5^ CFU/mL in PPLO medium. The diluted bacterial suspensions were inoculated into fresh PPLO medium at the ratio of 1:10 and incubated at 37 °C with 5% CO_2_ for 72 h. Samples were collected every 12 h to determine colony counts. To evaluate the impact of varying K^+^ concentrations (ranging from 0 mM to 500 mM) on bacterial growth, WT, cdaM*, and cdaM*::p*cdaM* strains (each at 10^5^ CFU/mL) were inoculated into PPLO medium supplemented with the respective KCl concentrations. After incubation under the same conditions for 12 h, colony counts were determined.

### Co-cultivation of *Mycoplasma bovis* with EBL cells

EBL cells at a concentration of 4 × 10^4^ cells/mL were seeded into each well of a 24-well plate. The *M. bovis* strains were inoculated onto EBL cells at a multiplicity of infection (MOI) of 0.5 and incubated at 37 °C in a CO_2_ incubator for 0, 24, 48, and 72 h. Following the incubation, the infected cells were subjected to a freeze–thaw cycle (−80 °C to 37 °C) to induce cell lysis and release intracellular bacteria. The lysates were collected, and bacterial colony counts were determined for each time point. Each experiment was performed in triplicate.

### Antiserum development

Fifteen four-week-old BALB/c mice were obtained from the Hubei Provincial Center for Disease Control and Prevention (Wuhan, China) and randomly divided into three groups. Five mice per group were immunized with 100 μg of recombinant protein, thoroughly emulsified in an equal volume of Freund’s complete adjuvant (Sigma-Aldrich Corporation, Darmstadt, Germany) for the initial immunization. Freund’s incomplete adjuvant (Sigma-Aldrich Corporation, Darmstadt, Germany) was used for subsequent immunizations. A control group was included under identical conditions, except that PBS was injected. Immunizations were administered via subcutaneous injection at 2-week intervals. One week following the last immunization, serum samples were collected from the mice.

### Western blot assay

The *M. bovis* cells were collected by centrifugation (8,000 g, 10 min), and proteins were extracted using RIPA lysis buffer (Sigma-Aldrich, St. Louis, MO, United States) containing protease inhibitors (Roche, China). Equal amounts of proteins were separated by SDS-PAGE and transferred onto a PVDF membrane (Burlington, MA, USA). The membranes were blocked with 5% (w/v) skimmed milk at room temperature for 3 h, and subsequently incubated overnight at 4 °C with primary antibodies, including mouse anti-MbovP579, a constitutively expressed protein used as an internal control and anti-MbovP496 (CdaM). After three washes with TBST buffer, a goat anti-mouse IgG antibody conjugated with horseradish peroxidase (HRP) (Abbkin, Wuhan, China) was used as the secondary antibody for subsequent detection. The enhanced chemiluminescence substrate kit (Thermo Fisher Scientific, MA, United States) was used to visualize the bands.

### RNA extraction and RT-qPCR

The wild type, cdaM*, and cdaM*::p*cdaM* strains were inoculated in 1 mL PPLO medium either with 50 mM K^+^ or without exogenous K^+^ for 12 h, and then collected by centrifugation at 1,000 × g for 10 min. Total RNA was isolated by using TRIzol^®^ reagent (Life Technologies, Carlsbad, CA, United States) and subsequently reverse transcribed into cDNA using the HiScript Q RT SuperMix (Vazyme, Nanjing, China). Quantitative real-time PCR (RT-qPCR) was carried out with SYBR Green Master Mix (Vazyme, Nanjing, China) on a ViiA^™^ 7 Real-Time PCR System (Applied Biosystems, Carlsbad, CA, United States). Each reaction was performed in triplicate, and the experiments were independently repeated three times. Relative gene expression was calculated using the $2^{–\Delta \Delta C_{T}}$ method, with normalization to *M. bovis* 16S rRNA levels.

### Pull down assay

MagStrep type2HC beads (IBA Lifesciences, Göttingen, Germany) were pre-coupled with 2.4 μM biotinylated c-di-AMP (BIOLOG Life Science Institute, Bremen, Germany) and incubated with 1.2 mg rTrkA in 1.5 mL of a PBS based buffer containing 10% (vol/vol) glycerol, 1 mM MgCl_2_, 5 mM Tris (pH 7.5), 230 mM NaCl, 0.5 mM DTT, 4 mM EDTA (pH 8.0), and 50 μg/mL BSA for 30 min at room temperature. The samples were washed four times with the same buffer excluding BSA at 2,000 × g and then resuspended in 50 μL SDS loading buffer. After boiling for 5 min, the beads were removed, and 18 μL of each sample was separated by SDS-PAGE [12% (wt/vol)] and stained with Coomassie Brilliant Blue (Biyuntian, Shanghai, China).

### ITC assay and BLI assay

The binding affinity of c-di-AMP and rTrkA was measured using a nano-ITC (TA, United States) in buffer (20 mM Tris–HCl, 500 mM NaCl, pH 6.0). Recombinant protein rTrkA and c-di-AMP were loaded into the reaction cell and syringe at concentrations of 20 μM and 400 μM, respectively. In each experimental trial, a total of 25 injections were performed, with each injection consisting of 2.5 μL. Thermodynamic parameters were calculated using NanoAnalyze software, and data analysis was conducted with the Origin 2018 software.

The rTrkA and its mutations rTrkA^R174A^, rTrkA^V180A^, rTrkA^V181A^, rTrkA^L191A^, rTrkA^P192A^, rTrkA^S193A^ and rTrkA^I207A^ were subjected to desalting using HiTrap desalting column (Cytiva, United States) via AKTA system (Cytiva, United States). Subsequently, the proteins were biotinylated using a biotinylation kit (Genemore, Jiangsu, China). The proteins (300 μg/mL) were immobilized on the streptavidin (SA) sensor, and binding kinetics were measured using different c-di-AMP concentrations (10, 25, 50, 100, 250, 500, 750, and 1,000 μM). The operational parameters were set as follows: a constant temperature of 30 °C was maintained, with a binding phase lasting 60 s and a subsequent dissociation phase extending for 90 s.

### Transcriptome sequencing analysis

The WT and cdaM* strains were cultured in 5 mL of PPLO medium with or without 50 mM K^+^ for 12 h. The bacterial pellets were collected by centrifugation (10,000 × g for 2 min) and washed three times with PBS. Subsequently, the pellets were rapidly frozen in nitrogen liquid and sent to Personal Biotechnology Co., Ltd. (Shanghai, China) for transcriptomic sequencing. Each treatment group contained three biological replicates. Differentially expressed genes (DEGs) were defined as significant at fold change ≥2 and *p* < 0.05.

### Measurement of intracellular K^+^ concentration

WT, cdaM*, and cdaM*::p*cdaM* strains were cultured in 20 mL PPLO medium with or without 50 mM K^+^ for 12 h, then the *M. bovis* cells were collected by centrifugation (2,200 × g for 10 min) and resuspended in 500 μL of ddH_2_O and lysed by sonication (50% power, 5 min working time, 3 s ultrasonic on-time, and 4 s off-time). The lysate was then centrifuged at 12,000 × g for 10 min at 25 °C, and the supernatant was adjusted to a final volume of 2 mL with ddH_2_O. The K^+^ concentration in *M. bovis* was measured using a potassium-specific lamp set to a wavelength of 766.5 nm on a flame atomic absorption spectrophotometer (Beijing Puxi General Instrument Co., Ltd., Beijing, China).

### Statistical analyses

All results were presented as the mean ± standard error of the mean (SEM). Differences in gene expression fold changes were evaluated using a two-tailed Student’s *t*-test. Cell colony counts and *M. bovis* growth curves were analyzed using Two-way ANOVA and the significance among groups was evaluated using *post-hoc* test (Tukey’s test) in GraphPad Prism 9 software (GraphPad Software, La Jolla, CA, United States). For the analysis of non-parametric data related to gene enrichment, fold-change calculations were combined with the false discovery rate (FDR) method. Significance values were presented as *p < 0.05, **p < 0.01, ****p < 0.001 and “ns” represented non-significance (*p* > 0.05).

## Results

### The DGA and RHR motifs of CdaM are necessary for its c-di-AMP cyclase activity

**Figure 1:**
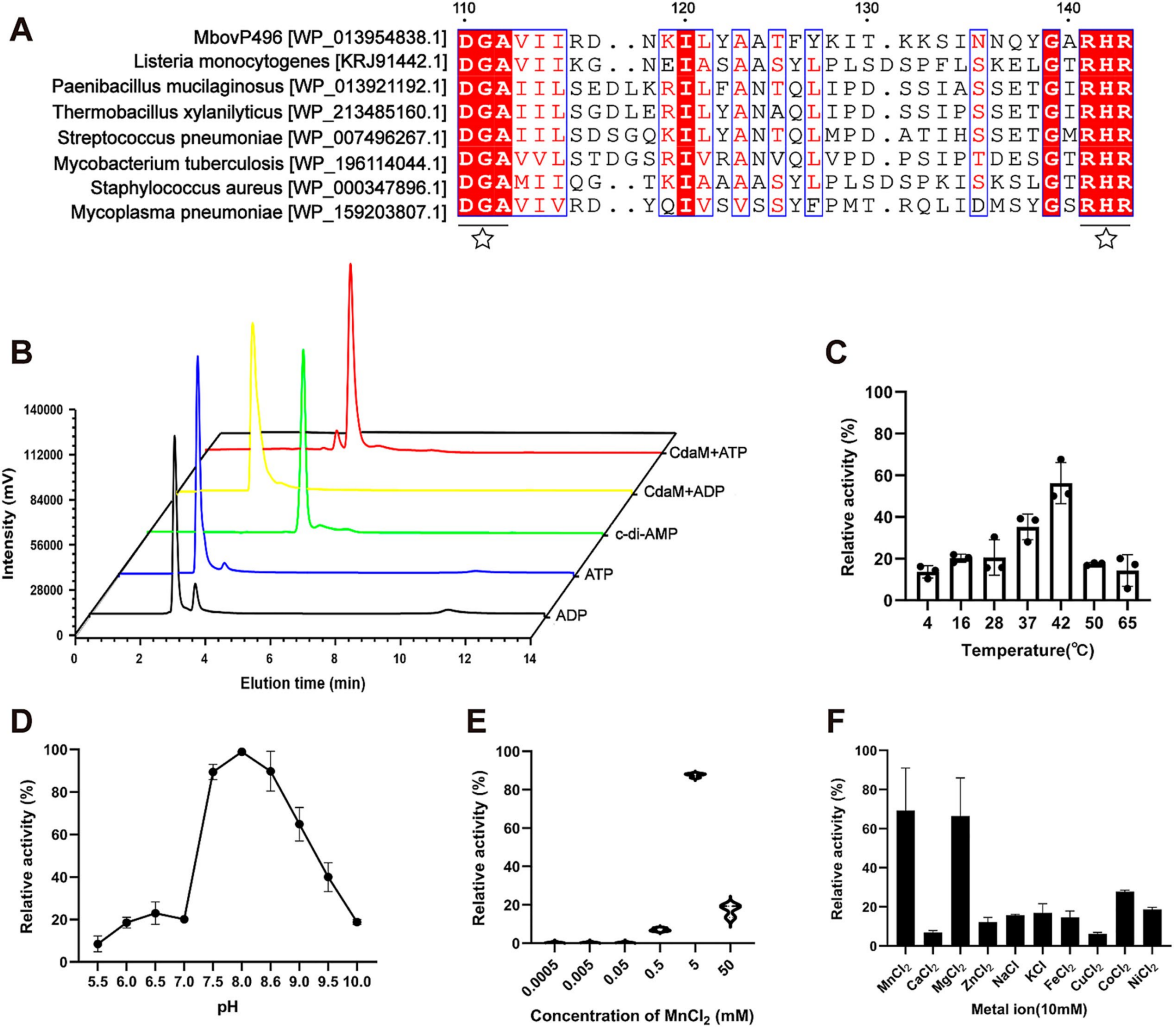
CdaM possesses c-di-AMP cyclase activity. **(A)** Alignment of conserved amino acids between CdaM and DACs in other bacteria. The conserved amino acid residues were marked with black five-pointed stars. **(B)** Confirmation of the c-di-AMP cyclase function and specific substrate for CdaM by HPLC. Black line represents the ADP standard sample, blue line represents the ATP standard sample, green line represents the c-di-AMP standard sample, yellow and red lines represent the enzymatic products obtained using ADP and ATP as substrates, respectively. **(C–F)** Investigation of the optimal enzymatic activity conditions for c-di-AMP cyclase CdaM. Relative activity of CdaM under different conditions: temperature **(C)**, pH **(D)**, mental ions **(E)**, and different concentration of Mn^2+^ **(F)**. Data represent the mean ± SEM from three independent biological experiments.

CdaM shares global similarity in different species (Supplementary Table S2) and possesses a transmembrane domain and an intracellular catalytic domain, which are typical features of the c-di-AMP cyclases within the DisA_N superfamily. Additionally, ClustalW multiple sequence alignment of representative members from the DisA_N superfamily revealed that the DGA and RHR motifs are highly conserved motifs (Figure 1A). The DisA_N superfamily encompasses a large group of diadenylate cyclases with substrate specificity, typically using ADP and ATP as substrates. To assess the enzymatic activity of CdaM, we produced His-tagged recombinant rCdaM proteins and tested their c-di-AMP synthesis activities. High-performance liquid chromatography (HPLC) analysis demonstrated that rCdaM induced the conversion of ATP into c-di-AMP, but not ADP (Figure 1B). The results indicated that CdaM is a c-di-AMP cyclase with specificity for ATP as a substrate. Additionally, the optimal enzymatic activity of CdaM was observed within the temperature range of 37–42 °C, at pH 7.5–8.6, and in the presence of Mn^2+^ or Mg^2+^, particularly with the optimal concentration of Mn^2+^ of 5 mM (Figures 1C–F). These results indicate that CdaM functions as a mesophilic and mildly alkaliphilic c-di-AMP cyclase that relies on Mn^2+^ or Mg^2+^ as essential metal ion cofactors.

**Figure 2:**
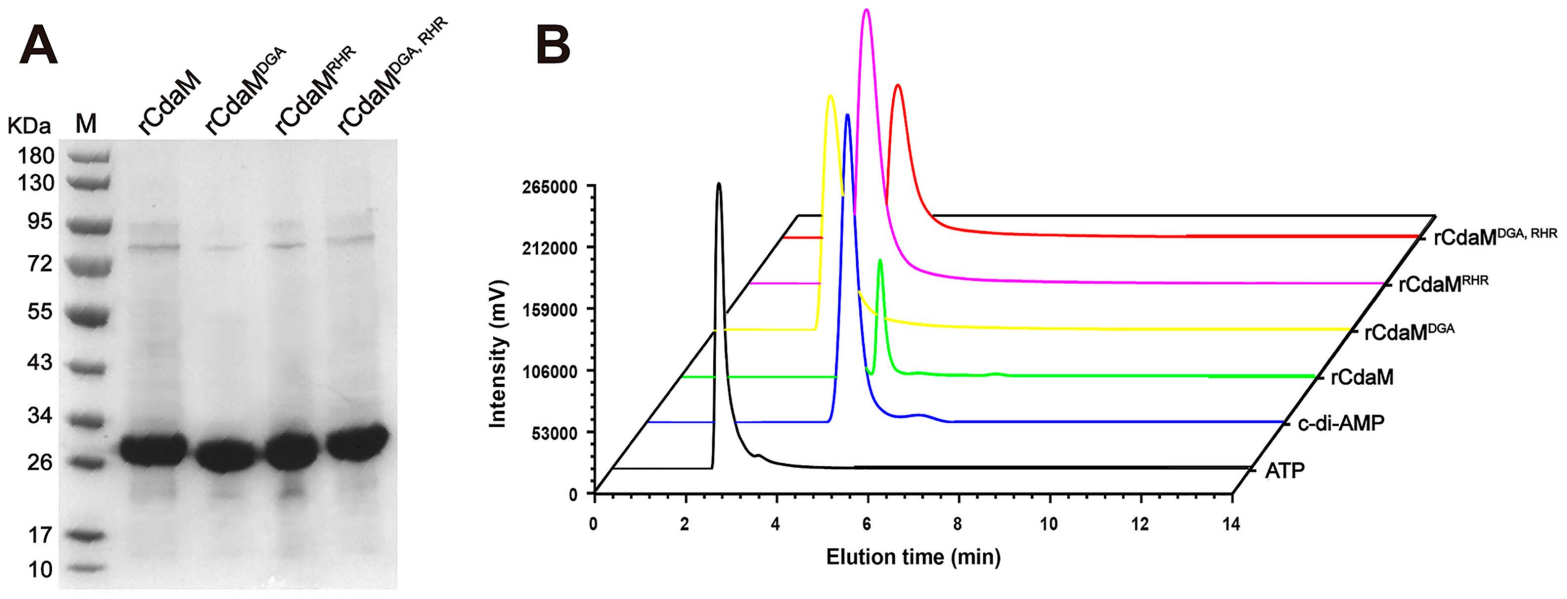
Essential motifs DGA and RHR in rCdaM for efficient c-di-AMP cyclase activity. **(A)** SDS-PAGE analysis of purified rCdaM and its mutant proteins rCdaM^DGA^, rCdaM^RHR^, and rCdaM^DGA, RHR^. **(B)** Confirmation of rCdaM functional sites using its mutant proteins rCdaM^DGA^ (yellow line), rCdaM^RHR^ (pink line), and rCdaM^DGA, RHR^ (red line), compared to rCdaM (green line). Black and blue lines represent ATP and c-di-AMP standard samples, respectively. rCdaM^DGA^, rCdaM^RHR^, and rCdaM^DGA, RHR^ represent: rCdaM^D110A/G111A^, rCdaM^R141A/H142A/R143A^, and rCdaM^D110A/G111A/R141A/H142A/R143A mutants^, respectively. The data represents the average of three independent experiments.

Furthermore, we successfully constructed and purified three mutants of rCdaM, including rCdaM^D110A/G111A^, rCdaM^R141A/H142A/R143A^, rCdaM^D110A/G111A/R141A/H142A/R143A mutants^, all of which had site-specific amino acid residues replaced with alanine (Figure 2A). HPLC analysis indicated that all the mutants lost the ability to synthesize c-di-AMP in the presence of ATP (Figure 2B). These results unequivocally demonstrated that the conserved motifs, DGA and RHR, are essential for CdaM c-di-AMP cyclase activity.

### The cdaM* mutant exhibits growth inhibition under high K^+^ conditions

**Figure 3:**
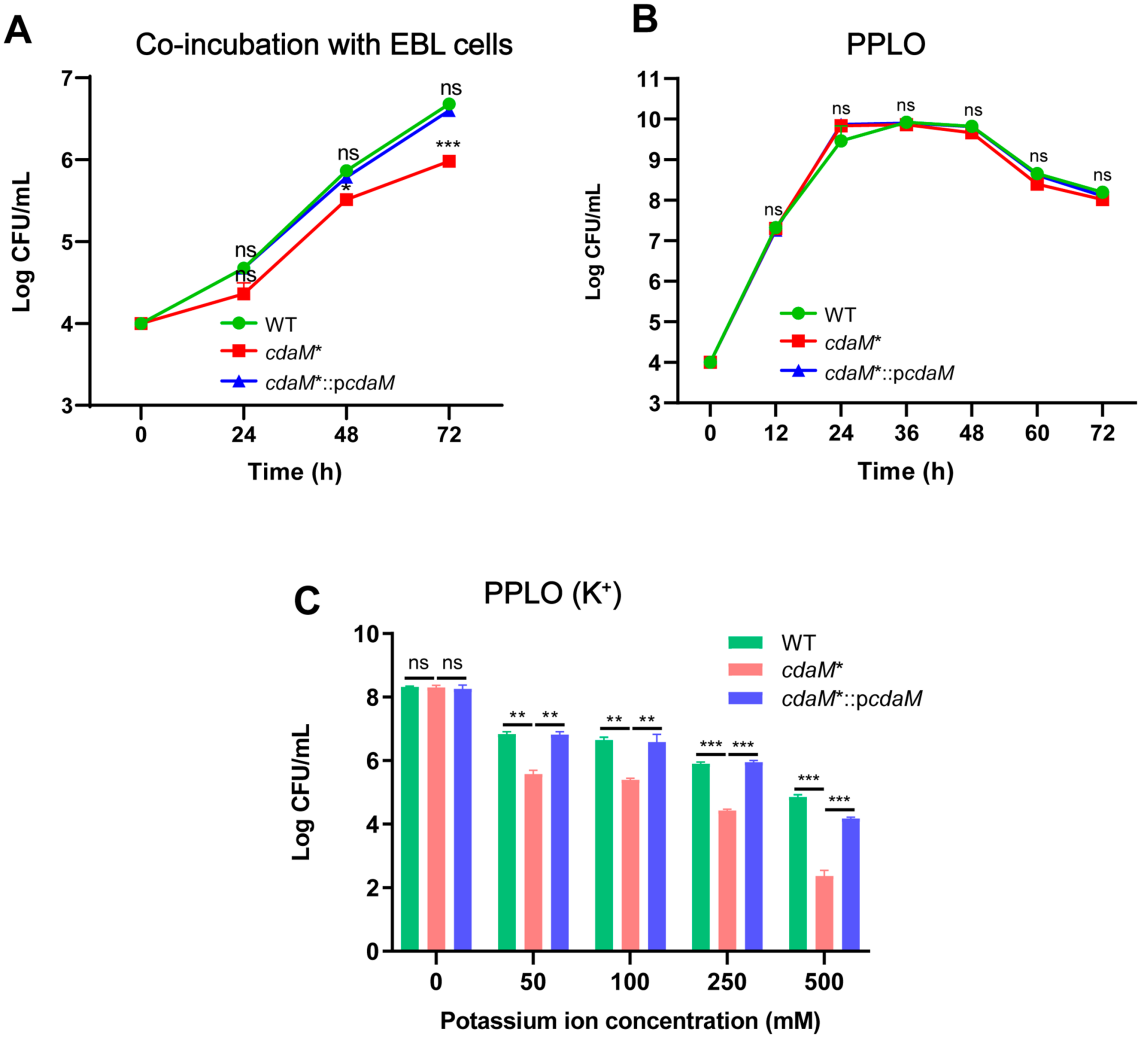
CdaM knockout mutants inhibit *M. bovis* growth **(A–C)** Growth of the wild-type strain (WT), mutant strain cdaM* (T5.415), and complementary strain cdaM*::p*cdaM* (CT5.415) under co-incubation with EBL cells **(A)**, PPLO medium **(B)**, and PPLO medium with increasing concentration of K^+^ **(C)**. Standard deviations are indicated by error bars. ***p < 0.001; **p < 0.01; ns = *p* > 0.05.

To study the role of CdaM in osmoadaptation, we compared the growth of *M. bovis* HB0801 (WT), cdaM* mutant (T5.415) and complemented strain cdaM*::p*cdaM* strains (CT5.415). Western blotting confirmed the absence of CdaM in cdaM* and its restoration in the complemented strain (Supplementary Figure S1). Because intracellular K^+^ levels are markedly higher than those in the extracellular milieu (Zacchia et al., 2016), and *M. bovis* primarily adheres to host cell surface and can subsequently invade them (Burki et al., 2015), we co-inoculated these strains with EBL cells to assess bacterial growth under K^+^ rich conditions. After 72 h of co-incubation, the cdaM* mutant exhibited significant growth inhibition compared with both the WT and the complemented strains (Figure 3A). In contrast, when the strains were cultured in PPLO medium (baseline of K^+^ ≈ 4 mM) without additional K^+^, no significant differences in growth were observed (Figure 3B), indicating that the growth defect of cdaM* is specifically associated with elevated K^+^ conditions. To further evaluate the K^+^ dependence of this phenotype, we performed growth assays in PPLO medium supplemented with increasing concentrations of KCl. Consistently, the cdaM* mutant exhibited significant reduced growth compared to both the WT and complemented strains in a K^+^-dependent manner (Figure 3C). Specifically, supplementation with 50 mM K^+^ was sufficient to reveal a clear growth defect in cdaM*, and complementation restored growth to levels comparable to WT (Figure 3C). Taken together, these findings suggest that the absence of CdaM (MbovP496), and consequently the reduction of c-di-AMP, is associated with increased expression of the K^+^ uptake transporter MbovP421 under high K^+^ stress.

### The cdaM* enhances K^+^ uptake transporter expression in high K^+^ environment

**Figure 4:**
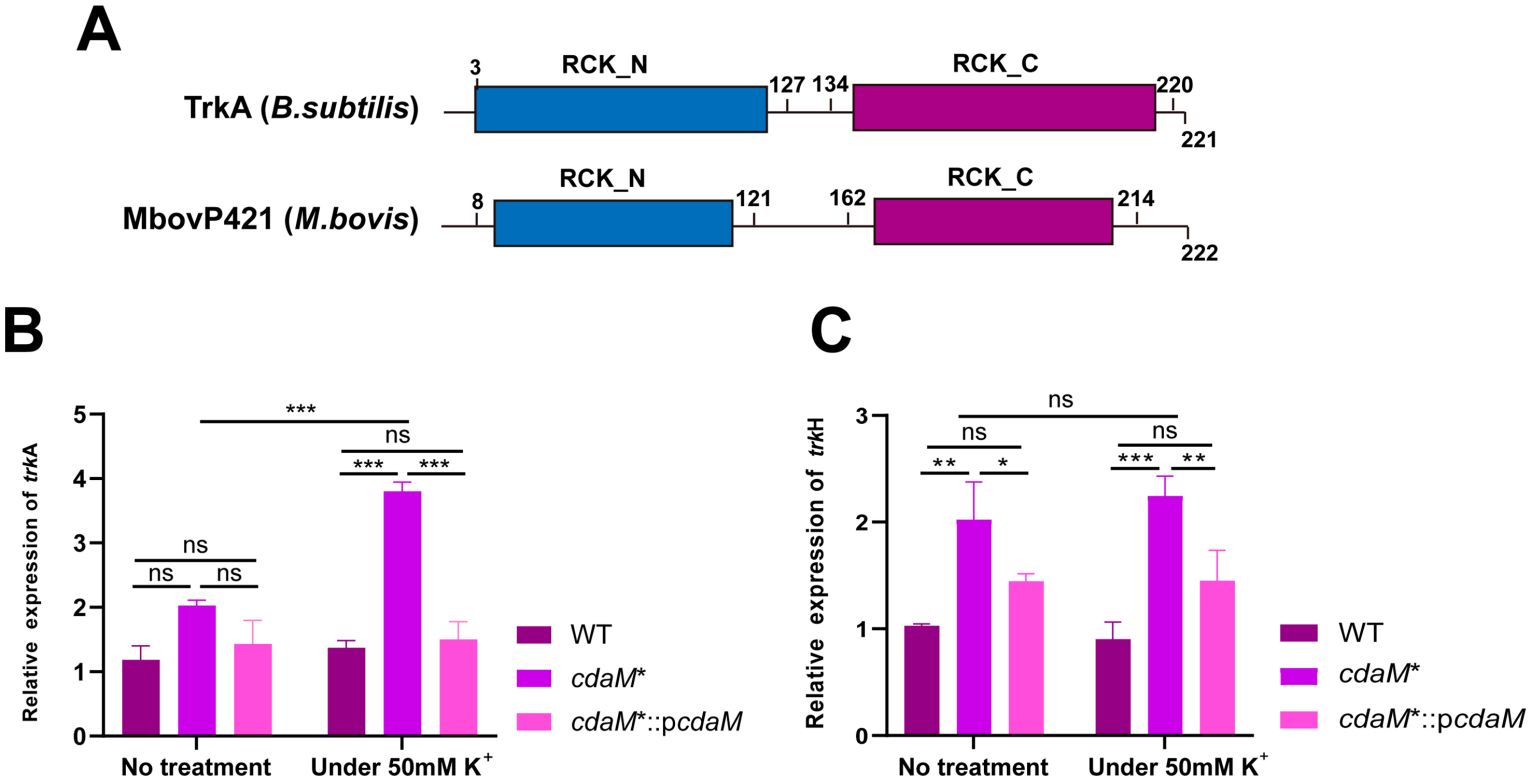
CdaM knockout mutants enhance the expression of K^+^ transporters under K^+^ stress. **(A)** Schematic diagram of typical TrkA from *B. subtilis* and MbovP421 from *M. bovis*, illustrating their conserved RCK_N and RCK_C domains. **(B,C)** RT-qPCR was performed to analyze the relative expression level of *trkA* (*Mbov_0421*) **(B)** and *trkH* (*Mbov_0415*) **(C)**. Standard deviations are indicated by error bars. ***p < 0.001; **p < 0.01; ns = *p* > 0.05.

Due to the high sensitivity to K^+^ and the growth inhibition phenotype observed in CdaM knockout strain cdaM*, we speculated that it might be attributed to the dysfunction of intracellular K^+^ transporter proteins. To further elucidate the reduced tolerance of the cdaM* to K^+^, we conducted a homology analysis of K^+^ transporters. The results showed that MbovP421 and MbovP415 of *M. bovis* shared homology with TrkA and TrkH family members, respectively. Among them, MbovP421, along with *B. subtilis*, possess the conserved RCK_N and RCK_C domains (Figure 4A), whereas MbovP415, similar to most TrkH proteins, contains multiple transmembrane helices (Supplementary Figure S2). Accordingly, MbovP421 and MbovP415 are hereafter referred to as TrkA and TrkH, respectively. In addition, compared to the untreated group, the cdaM* exhibited a 1.88-fold upregulation of *trkA* at the mRNA level under 50 mM K^+^ conditions (Figure 4B). Meanwhile, RT-qPCR also showed that the expression of *trkH* was up-regulated in the cdaM* compared to both WT and cdaM*::p*cdaM*. However, no significant up-regulation of *trkH* was observed in the cdaM* between untreated group and 50 mM K^+^ condition (Figure 4C). Taking together, these findings suggest that the absent of CdaM enhances the expression of the K^+^ transporters *trkA* in a high K^+^ environment.

### K^+^ transporter TrkA binds to c-di-AMP

**Figure 5:**
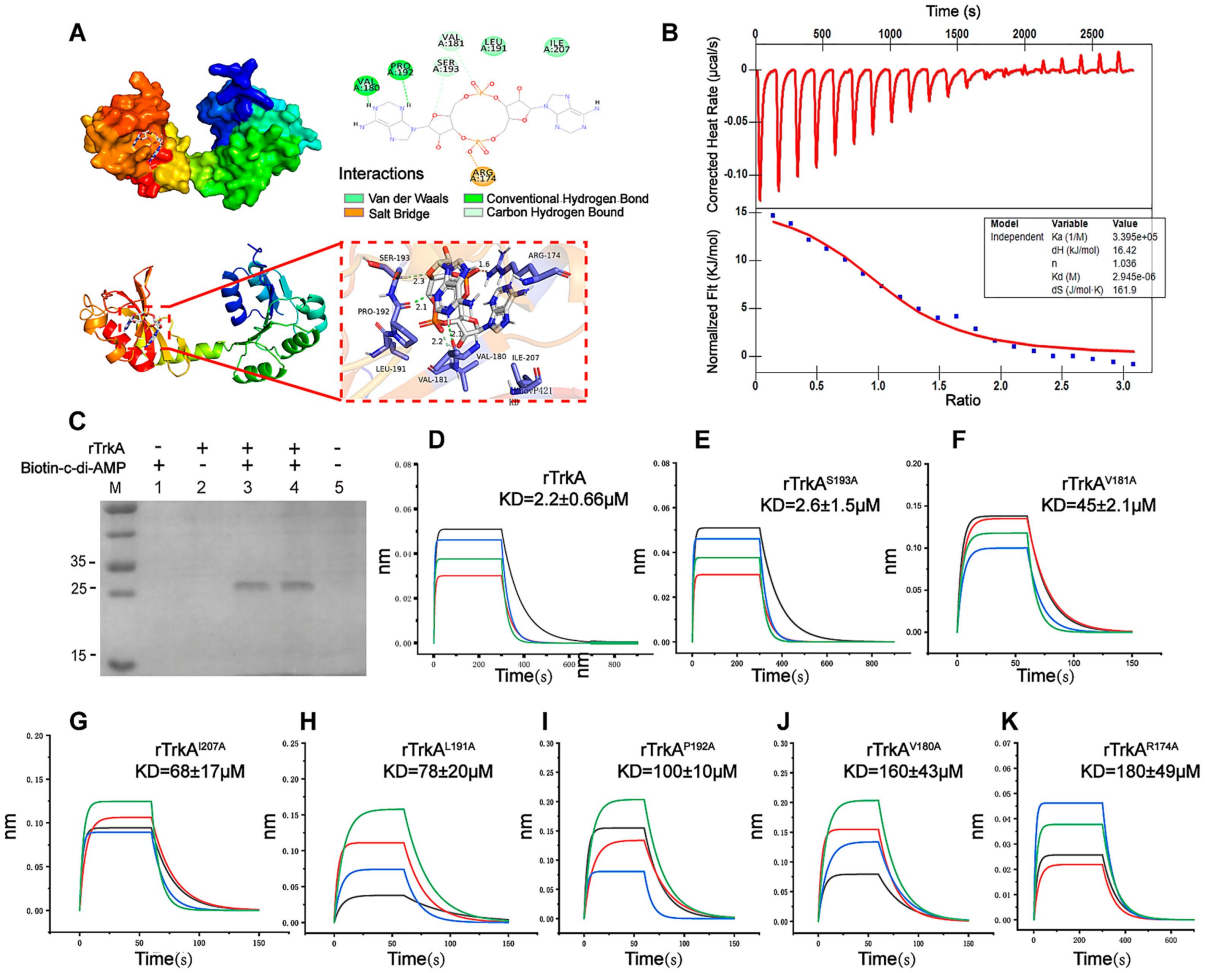
Potassium transporter rTrkA can bind to c-di-AMP. **(A)** Molecular docking predicts multiple potential binding sites for c-di-AMP on TrkA. The protein backbone is represented as a cartoon, with the ligand (carbon in magenta) and active site residues (carbon in blue) shown in stick representation. Hydrogen bonds are indicated by dashed lines. **(B–D)** Verification of the interaction between rTrkA and c-di-AMP via ITC **(B)**, pull-down **(C)**, and BLI **(D)**. **(E–K)** Confirmation of the interaction between rTrkA mutants and c-di-AMP via BLI. rTrkA mutants include rTrkA^S193A^ **(E)**, rTrkA^V181A^ **(F)**, rTrkA^I207A^ **(G)**, rTrkA^L191A^ **(H)**, rTrkA^P192A^ **(I)**, rTrkA^V180A^ **(J)**, rTrkA^R174A^ **(K)**.

Recent studies have revealed that cytoplasmic c-di-AMP binds to TrkA in bacteria, thereby inhibiting its interaction with TrkH and modulating K^+^ uptake, which is normally promoted by TrkA-TrkH complex formation (Corratgé-Faillie et al., 2010; Tanudjaja et al., 2023). To investigate whether a similar regulatory mechanism operates in *M. bovis*, we focused on the TrkA homolog encoded by MbovP421 (hereafter referred to as TrkA). A three-dimensional structural model of *M. bovis* TrkA was generated using AlphaFold. c-di-AMP was subsequently docked into the simulated structure to assess potential binding interfaces and interaction modes. The conformation with the lowest binding energy (−8.8 kcal/mol) indicated that c-di-AMP interacts with TrkA via hydrogen bonds, ionic bonds, van der Waals forces and hydrophobic forces (Figure 5A). To evaluate the binding mode of the complex, we purified the His-tagged recombinant TrkA (rTrkA), and molecular interaction assays were performed using isothermal titration calorimetry (ITC), pull-down assays, and bio-layer interferometry (BLI) assays. All three experiments confirmed the interactions between rTrkA and c-di-AMP (Figures 5B–D). To further investigate the interaction sites of TrkA, we constructed seven-point mutant proteins: rTrkA^R174A^, rTrkA^V180A^, rTrkA^V181A^, rTrkA^L191A^, rTrkA^P192A^, rTrkA^S193A^ and rTrkA^I207A^ (Supplementary Figure S3), and utilized BLI assays to analyze the interactions between these mutant proteins and c-di-AMP (Figures 5E–K). The results revealed a significant increase in KD values at all mutant sites, except for the S193A residue (Figures 5E–K). Notably, the increases were particularly pronounced at R174A, V180A, and P192A, with the KD values exceeding a 40-fold enhancement (Figures 5I–K). Therefore, these findings confirm the interaction between rTrkA and c-di-AMP.

### CdaM plays a role in transport activity and pyruvate metabolism

**Figure 6:**
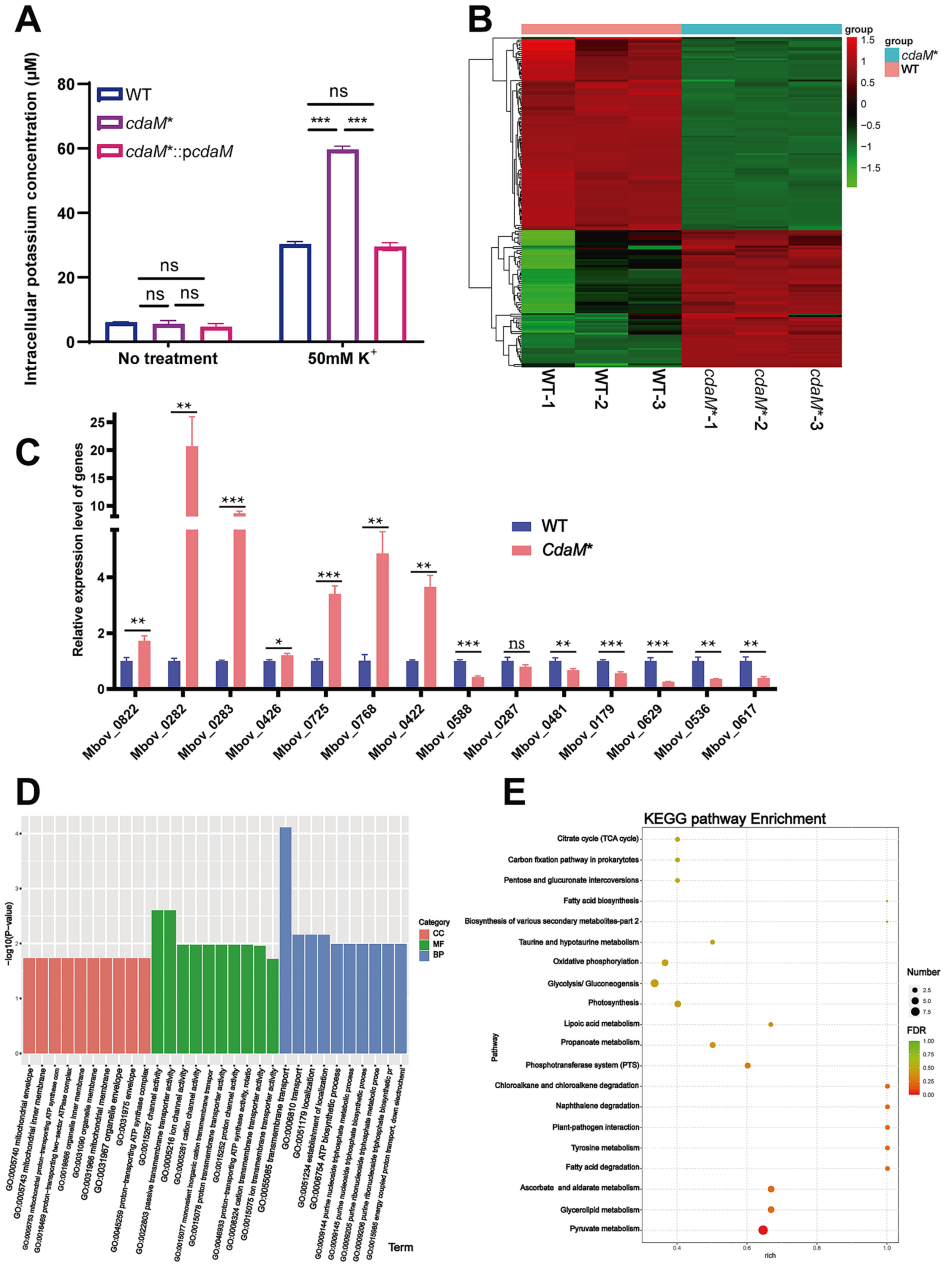
Transcriptomics analysis of the DEGs in cdaM* compared to WT under 50 mM K^+^ treatment. **(A)** Detection of intracellular K^+^ levels in WT, cdaM*, and cdaM*::*pcdaM* strains with or without addition of 50 mM K^+^. **(B)** Heatmap of DEGs between the cdaM* and wild type strain (WT) (difference ≥1.5-folds; *p* < 0.05). Transcriptomic analyses were performed in triplicate for each strain. In the heatmap, each column corresponds to an individual replicate, while each row represents a specific gene. Red and blue indicate relatively upregulated and downregulated differentially expressed genes (DEGs), respectively. The dendrograms displayed above and to the left of the heatmap depict the clustering of samples and genes, respectively. **(C)** The relative expression levels of DEGs in *M. bovis* HB0801 WT and cdaM* strains were further validated by RT-qPCR. Error bars represent standard deviations, and statistical significance is indicated by asterisks (*p < 0.05, **p < 0.01, ***p < 0.001, ns = *p* > 0.05). **(D)** Histogram of GO enrichment analysis of DEGs. The −log_10_ (*p*-value) range is from 0 to 4. Red represents cellular component (CC), green represents molecular function (MF) and blue represents biological process (BP) differences between cdaM* and WT. **(E)** Bubble chart of Kyoto Encyclopedia of Genes and Genomes (KEGG) pathway analysis. The *y*-axis shows the different KEGG pathways, and the *x*-axis represents the gene ratio, which indicates the ratio of the number of DEGs to the total number of annotated genes in this pathway. The size of the dot correlates with the number of DEGs annotated in the pathway.

The above data illustrate that cdaM* increased K^+^ transporter expression and exhibited growth inhibition. To investigate the dynamics of c-di-AMP on intracellular K^+^ concentration, we measured the intracellular K^+^ levels using flame atomic absorption spectrometry (FAAS). The results revealed that the intracellular K^+^ concentration in cdaM* was increased 2-fold under the 50 mM K^+^ condition compared with that in WT and complemented strain (Figure 6A). In addition, RNA-seq was employed to analyze the gene expression profile of cdaM* and WT under 50 mM K^+^ for 12 h. Differential gene expression analysis revealed 171 genes with significant alterations in cdaM*, of which 71 were up-regulated and 100 were down-regulated (Figure 6B and Supplementary Table S3). Subsequently, RT-qPCR analysis further confirmed changes in mRNA levels for 13 out of the 14 selected genes, including 7 up-regulated genes and 6 down-regulated genes, which were consistent with the RNA-seq data (Figure 6C). Furthermore, Gene Ontology (GO) enrichment analysis showed that the differentially expressed genes (DEGs) were mainly associated with transmembrane transport activity, both in terms of molecular function and biological processes (Figure 6D). Consistent with our findings, the K^+^ transporters *trkA* (FoldChange = 1.74) was specifically up-regulated in the cdaM* strain. Kyoto Encyclopedia of Genes and Genomes (KEGG) analysis identified that DEGs related to genetic information processing were notably affected, along with microbial metabolism in various settings, pyruvate and glycerolipid metabolism, ascorbate and aldarate metabolism, glycolysis, and production of secondary metabolites (Figure 6E). Among these, 14 genes in the cdaM* strain were specifically up-regulated, relating to pyruvate metabolism. Additionally, three up-regulated genes, Mbov_0161, Mbov_0434, and Mbov_0162, were involved in the glycerolipid metabolism pathway. Four down-regulated genes, Mbov_0720, Mbov_0723, Mbov_0721, and Mbov_0719, are involved in the metabolic pathway of ascorbate and aldarate. These transcriptome data underscore the pivotal regulatory role of several metabolic pathways in maintaining intracellular homeostasis and responding to altered K^+^ condition, further evidenced by the observed growth inhibition in cdaM* under 50 mM K^+^ condition.

## Discussion

Since bacteria are much smaller than complex eukaryotic organisms, they are more easily exposed to the constantly changing external environments. To achieve coordinated regulation of various physiological processes, bacteria must continuously sense external signals (Orr et al., 2016). Second messengers, such as cyclic nucleotides, play a central role in this coordination by linking environmental sensing to downstream regulatory networks (Hengge et al., 2019; Nelson and Breaker, 2017).

Given that c-di-AMP cyclases are conserved in most bacteria, we decided to study their function in the smallest bacteria, *Mycoplasma*. However, signaling pathways within *Mycoplasma* are rarely reported. Using *M. bovis* as a model, our results revealed that c-di-AMP functions as an important second messenger in *M. bovis*. Most bacteria contain only one cyclic diadenylate cyclase, typically of the CdaA type (Rismondo et al., 2016), while some strains, like *Bacillus subtilis* and its proximal strains, contain three diadenylate cyclases (Gundlach et al., 2015). In this study, we identified a c-di-AMP cyclase in *M. bovis*, designated CdaM (MbovP496), which is homologous to the CdaM (MPN244) in *M. pneumoniae* (Blötz et al., 2017). Diadenylate cyclases (DACs) are generally known to synthesize c-di-AMP through a condensation reaction using ATP or ADP. Notably, our biochemical analyses revealed that *M. bovis* CdaM utilizes ATP exclusively for c-di-AMP synthesis, and that its catalytic activity critically depends on the conserved DGA and RHR motifs. These features are consistent with previous reports (Du and Sun, 2015; Pham et al., 2016).

Previous studies have shown that disruption of c-di-AMP can directly lead to growth defects under low osmolarity or pyruvate-deficient media (Jackson-Litteken et al., 2021), demonstrated that the balance of c-di-AMP plays an important role in intracellular K^+^ homeostasis (Cereija et al., 2021). However, the specific regulatory mechanisms in *Mycoplasma* species remained largely unexplored. Through homologous analysis, we identified two components of Trk potassium uptake system in *M. bovis*. *Mbov_0421*, encoding the cytoplasmic regulatory subunit TrkA, and *Mbov_0415*, encoding the multi-transmembrane channel protein TrkH. We further demonstrated that c-di-AMP directly binds to *M. bovis* TrkA via its RCK_C domain, with residues R174, V180, and P192 playing critical roles in this interaction. This is consistent with observations in other bacterial systems (Corrigan et al., 2013; Kim et al., 2015). Deletion of the c-di-AMP cyclase CdaM abolishes this regulatory constraint, leading to dysregulated K^+^ uptake. Under elevated extracellular K^+^ conditions, loss of c-di-AMP-mediated inhibition of TrkA results in excessive K^+^ influx, disruption of osmotic homeostasis, and pronounced growth inhibition of the *cdaM*^⁎^ mutant. Meanwhile, we observed increased transcriptional expression of TrkA in the cdaM* strain, suggesting that c-di-AMP may additionally suppress TrkA expression, thereby limiting K^+^ uptake. This mode of regulation would be distinct from the canonical model in which c-di-AMP competitively binds to TrkA to block its interaction with TrkH (Bai et al., 2014; Zarrella et al., 2020). Given that c-di-AMP-responsive riboswitch have been reported to regulate K^+^ transporter expression via the 5′ untranslated region (Gundlach et al., 2017b; Nelson et al., 2013), we speculate that a similar regulatory mechanism may operate in *M. bovis*, although direct experimental validation will be required.

**Figure 7:**
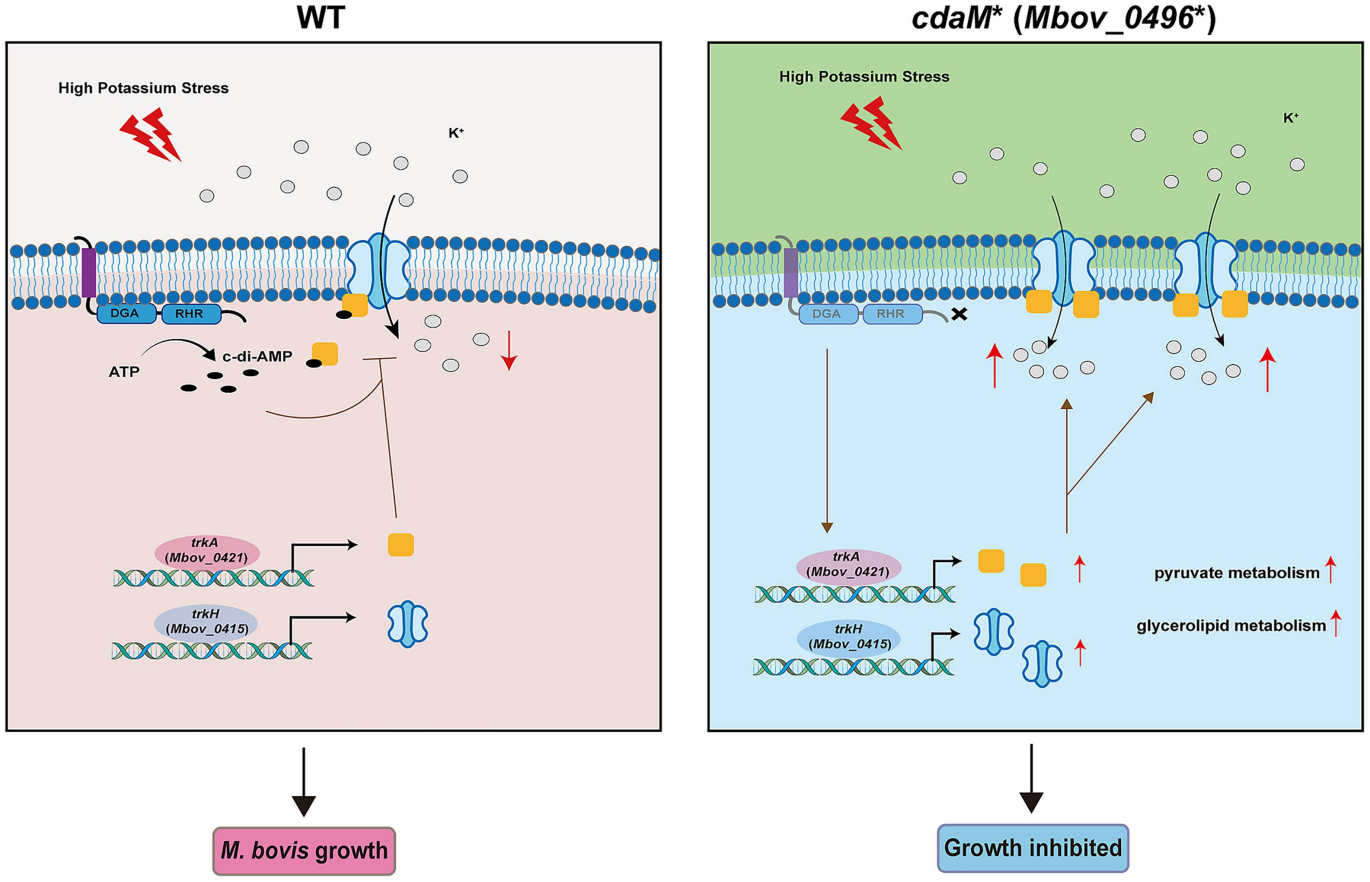
Diagram of the regulatory mechanism of CdaM under K^+^ stress. In the WT strain, CdaM normally produces c-di-AMP, which binds to TrkA and modulates another K^+^ transporter, TrkH to restrict K^+^ influx, sustaining *M. bovis* growth in high K^+^. In the *cdaM* mutant, dysfunction of CdaM triggers up-regulation of K^+^ transporters, exacerbating K^+^ influx and inhibiting *M. bovis* growth. Concomitantly, intracellular K^+^ imbalance is associated with enhanced pyruvate metabolism and glycerolipid metabolic activity, likely reflecting compensatory metabolic reprogramming in response to ionic stress.

Based on these findings, we propose a schematic model (Figure 7) in which CdaM-derived c-di-AMP functions as a central integrator linking extracellular potassium availability to K^+^ transporter activity, metabolic adaptation, and growth fitness in *M. bovis*. In this model, elevated extracellular K^+^ stimulates c-di-AMP signaling, which restrains K^+^ influx through TrkA and preserves ionic homeostasis. Disruption of this pathway in the *cdaM*^⁎^ mutant leads to intracellular K^+^ accumulation and ionic stress, which in turn triggers global metabolic reprogramming. Consistent with this model, transcriptomic analyses revealed that loss of CdaM broadly affects genes involved in transmembrane transport and central metabolism. Notably, pathways related to pyruvate metabolism, glycerolipid metabolism were significantly upregulated in the *cdaM*^⁎^ mutant. These metabolic changes likely represent compensatory responses aimed at maintaining energy production, membrane remodeling, and cellular viability under conditions of disturbed K^+^ homeostasis. Similar links between c-di-AMP signaling, potassium regulation, and central carbon metabolism have been reported in other bacterial systems (Fahmi et al., 2019; Selim et al., 2021; Choi et al., 2017). While several metabolic pathways were altered in the absence of CdaM, whether targeted supplementation of specific metabolites can functionally rescue the growth defects observed under high K^+^ conditions warrants further investigation. Therefore, the DAC-catalyzed synthesis of c-di-AMP is tightly controlled to maintain optimal cellular levels, as either excess or deficiency in c-di-AMP can disrupt metabolic equilibrium and lead to growth defects (Ning et al., 2022). Overall, these findings highlight the complex interplay between second messenger signaling, ion homeostasis, and metabolic regulation in *M. bovis*.

In summary, we demonstrate that CdaM functions as a c-di-AMP cyclase that promotes the survival of *M. bovis* under high K^+^ stress by restraining K^+^ influx through the TrkA. Loss of CdaM disrupts this regulatory circuit by abolishing c-di-AMP-mediated inhibition of TrkA while simultaneously enhancing TrkA expression, leading to excessive K^+^ uptake, metabolic imbalance, and growth inhibition. Together, these findings reveal a c-di-AMP-dependent adaptive mechanism in *Mycoplasma* and highlight DisA_N superfamily proteins as promising targets for antimicrobial development.

## Data Availability

The RNA-seq data have been deposited in the Gene Expression Omnibus (GEO) database under accession code GSE282920.

## Footnotes

1
https://blast.ncbi.nlm.nih.gov/Blast.cgi?PAGE=Proteins

2
http://www.ncbi.nlm.nih.gov/Structure/cdd/wrpsb.cgi

3
https://www.genome.jp/tools-bin/clustalw

4
https://espript.ibcp.fr/ESPript/cgi-bin/ESPript.cgi

5
https://www.rcsb.org/

6
https://pubchem.ncbi.nlm.nih.gov/
