# Supplementary material for: Regulation of potassium homeostasis in Mycoplasma bovis by the diadenylate cyclase CdaM
Source: Front Microbiol. 2026 Mar 13;17:1757129. doi: 10.3389/fmicb.2026.1757129 (PMC13022847; doi:10.3389/fmicb.2026.1757129)
Supplement: Supplementary file 1 [file Table_1.DOCX]

**Table S1 Oligo nucleotide primers used in this study**

| **Primers** | **Primer sequences** | **Product length** |
| --- | --- | --- |
| Mbov_0496-F | CGGGGTACCAAAAGCGAATTAATCTTGTTGATAGTAA | 579 |
| Mbov_0496-R | CCGGAATTCTTAGTCCTTTAGGTATTTAATTAATTGTTC |  |
| 0496dlt-F | CGGGGTACCATGAAAAGCGAAATTTCACTA | 510 |
| 0496dlt-R | CCGGAATTCTTAGTCCTTTAGGTATTTAATTAATTGTT |  |
| CT-0496-F | CGCGGATCCATGAAAAGCGAATTAATCTTGTTG | 652 |
| CT-0496-R | CATGCCATGGTTAGTCCTTTAGGTATTTAATTAATTGTTC |  |
| 0496dlt DGA-F1 | CGCGGATCCATGAAAAGCGAAATTTCACTATTC | 269 |
| 0496dlt DGA-R1 | ACTGCTCCTGCATGCAAAGGAGAATACTTG |  |
| 0496dlt DGA-F2 | TTGCATGCAGGAGCAGTAATTATAAGAGAT | 258 |
| 0496dlt DGA-R2 | CCGGAATTCTTAGTCCTTTAGGTATTTAATTAATTGTTC |  |
| 0496dlt RHR-F1 | CGCGGATCCATGAAAAGCGAAATTTCACTATTC | 372 |
| 0496dlt RHR-R1 | ACCCATTGCGGCTGCGTGTGCTGCACCATATTGA |  |
| 0496dlt RHR-F2 | ATGGTGCAGCACACGCAGCCGCAATGGGTATAAGC | 167 |
| 0496dlt RHR-R2 | CCGGAATTCTTAGTCCTTTAGGTATTTAATTAATTGTTC |  |
| Mbov_0421-F | CGGGGTACCATGGCTAAAAAAAGAA | 681 |
| Mbov_0421-F | CCGGAATTCTTATTTATTTAGTTCGGCAAGC |  |
| 421-R174A-F | AGAAATTAAATTCGCAGACCTTGGGG | 681 |
| 421-R174A-R | CCGGAATTCTTATTTATTTAGTTCGGCAAGCA |  |
| 421-V180A-F | GAGACCTTGGGGTAAGTCGTGTTTTAATTAAAAGAG | 681 |
| 421-V180A-R | AATTAAAACACGACTTACCCCAAGGTCTCTGA |  |
| 421-V181A-F | CAGAGACCTTGGGGTAAGTGTTCGTTTAATTA | 681 |
| 421-V181A-R | GCACCTCTTTTAATTAAACGAACACTTACCCCA |  |
| 421-L191A-F | GCATTCGGCCTAGCGGGCTAACA | 681 |
| 421-L191A-R | GCCCGCTAGGCCGAATGCTTC |  |
| 421-P192A-F | TTTTGCGTAGCGGGCTAACAAC | 681 |
| 421-P192A-R | AGCCCGCTACGCAAAATGCTTC |  |
| 421-S193A-F | GAAGCATTTTGCCTCGCGGGCTAA | 681 |
| 421-S193A-F | GTTGTTAGCCCGCGAGGCAA |  |
| 421-I207A-F | GGAGATTTACTTACCTTACGTGGTAAAGTTGAAGATG | 681 |
| 421-I207A-R | CTTCAACTTTACCACGTAAGGTAAGTAAATCTCCTC |  |

**Note: Restriction enzyme sites are underlined.**
