# Supplementary material for: Regulation of potassium homeostasis in Mycoplasma bovis by the diadenylate cyclase CdaM
Source: Front Microbiol. 2026 Mar 13;17:1757129. doi: 10.3389/fmicb.2026.1757129 (PMC13022847; doi:10.3389/fmicb.2026.1757129)
Supplement: Supplementary file 2 [file Table_2.DOCX]

**Table S2 Screening of c-di-AMP synthase in *M. bovis***

| **Bacteria name** | **Bacteria gene** | **Hit gene in *M. bovis*** | **Identity (%)** | **E value** |
| --- | --- | --- | --- | --- |
| *Bacillus subtilis* | O31854.3 | Mbov_0496 | 45.16 | 3e-33 |
| *Staphylococcus aureus* | QBS07837.1 | Mbov_0496 | 36.52 | 1e-31 |
| *Mycobacterium tuberculosis* | P9WNW5.1 | Mbov_0496 | 26.88 | 7e-05 |
| *Mycolicibacterium smegmatis* | 7Y0D_H | Mbov_0496 | 26.88 | 1e-04 |
| *Listeria monocytogenes* | KRJ91442.1 | Mbov_0496 | 42.21 | 6e-35 |
| *Lactococcus lactis* | KSU32270.1 | Mbov_0496 | 45.27 | 2e-36 |
| *Escherichia coli* | WGI63513.1 | Mbov_0496 | 28.95 | 0.009 |
| *Mycoplasmoides pneumoniae* | WP_159203807.1 | Mbov_0496 | 40.54 | 5e-35 |
| *Mycoplasmoides gallisepticum* | WP_085063791.1 | Mbov_0496 | 40.50 | 5e-26 |
| *Chlamydia trachomatis* | ROT60540.1 | Mbov_0496 | 36.20 | 1e-23 |
| *Thermovirga lienii* | AER66492.1 | Mbov_0496 | 36.36 | 6e-28 |
