# Supplementary figures and images for: Regulation of potassium homeostasis in Mycoplasma bovis by the diadenylate cyclase CdaM
Source: Front Microbiol. 2026 Mar 13;17:1757129. doi: 10.3389/fmicb.2026.1757129 (PMC13022847; doi:10.3389/fmicb.2026.1757129)

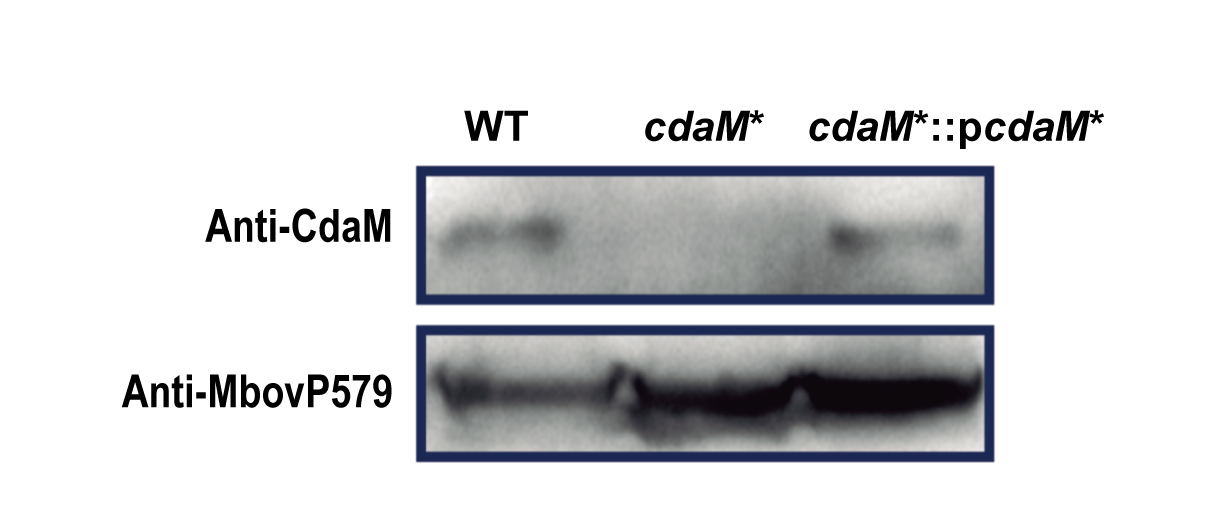

Supplement: Supplementary file 4 [file Image_1.tif]

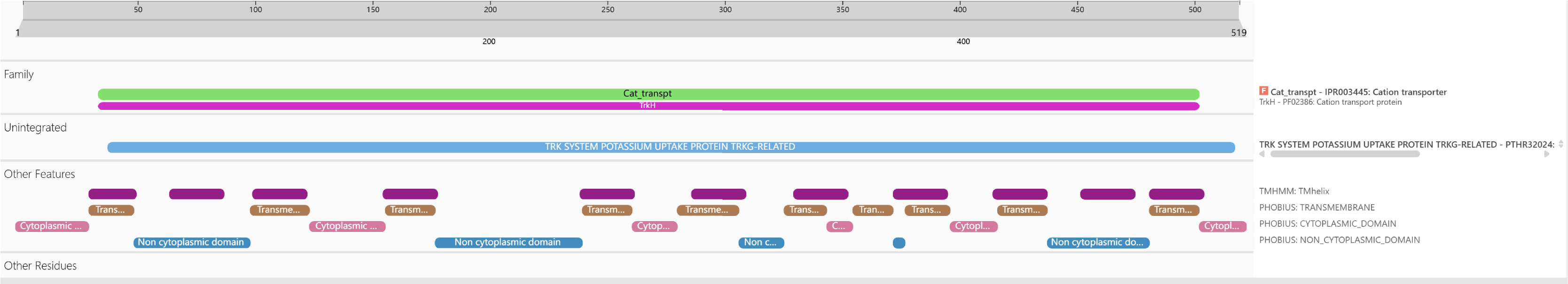

Supplement: Supplementary file 5 [file Image_2.tif]

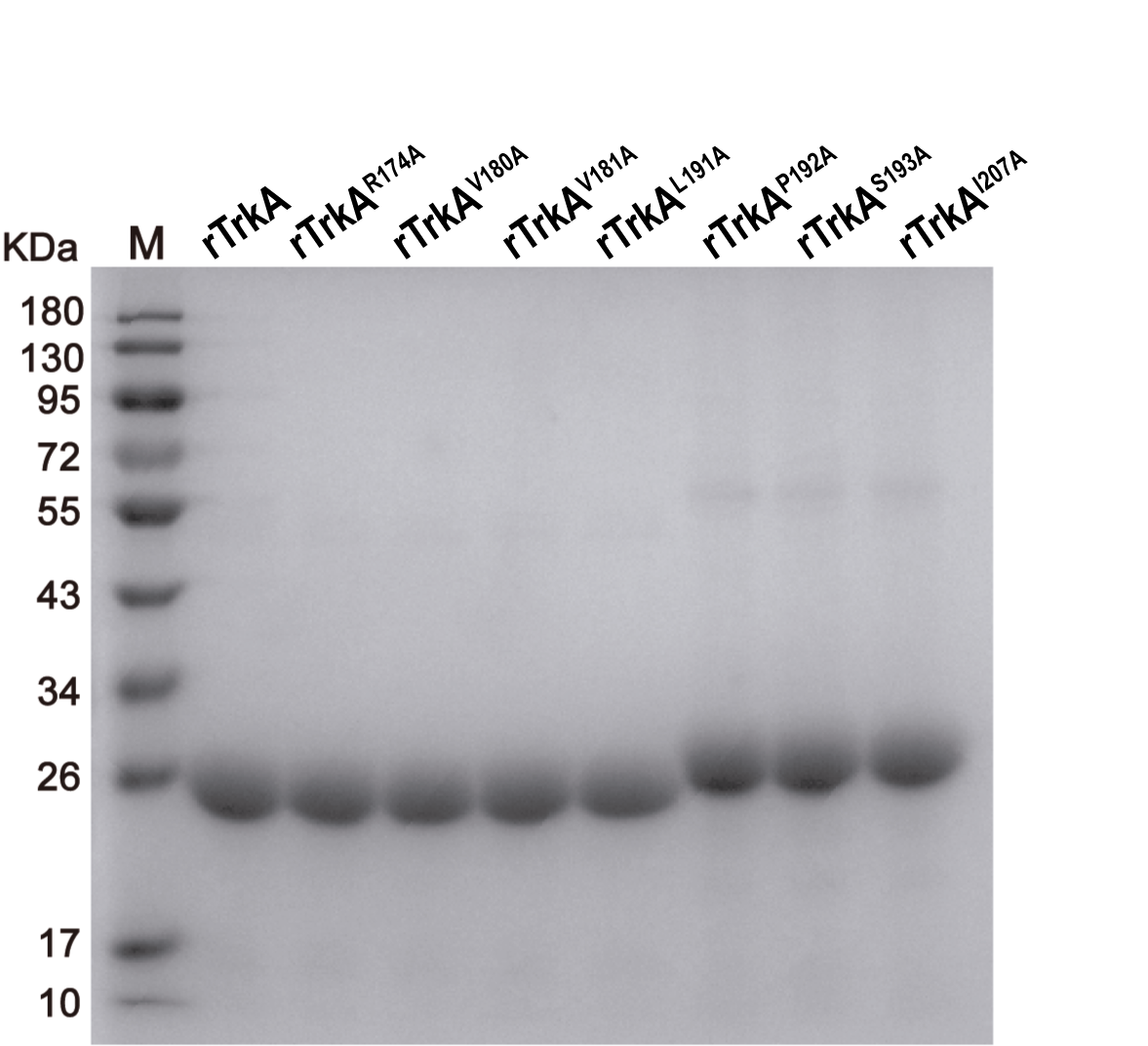

Supplement: Supplementary file 6 [file Image_3.tif]
